# Bottom-up GGM algorithm for constructing multilayered hierarchical gene regulatory networks that govern biological pathways or processes

**DOI:** 10.1186/s12859-016-0981-1

**Published:** 2016-03-18

**Authors:** Sapna Kumari, Wenping Deng, Chathura Gunasekara, Vincent Chiang, Huann-sheng Chen, Hao Ma, Xin Davis, Hairong Wei

**Affiliations:** School of Forest Resources and Environmental Science, Michigan Technological University, Houghton, MI 49931 USA; Department of Forestry and Environmental Resources, North Carolina State University, Raleigh, NC 27695 USA; Statistical Methodology and Applications Branch, Division of Cancer Control and Population Sciences, National Cancer Institute, National Institutes of Health, Rockville, MD 20850 USA; NCCWA, USDA ARS, Kearneysville, WV 25430 USA

**Keywords:** Multilayered gene regulatory network, Pathway, Microarray or RNA-seq data

## Abstract

**Background:**

Multilayered hierarchical gene regulatory networks (ML-hGRNs) are very important for understanding genetics regulation of biological pathways. However, there are currently no computational algorithms available for directly building ML-hGRNs that regulate biological pathways.

**Results:**

A bottom-up graphic Gaussian model (GGM) algorithm was developed for constructing ML-hGRN operating above a biological pathway using small- to medium-sized microarray or RNA-seq data sets. The algorithm first placed genes of a pathway at the bottom layer and began to construct a ML-hGRN by evaluating all combined triple genes: two pathway genes and one regulatory gene. The algorithm retained all triple genes where a regulatory gene significantly interfered two paired pathway genes. The regulatory genes with highest interference frequency were kept as the second layer and the number kept is based on an optimization function. Thereafter, the algorithm was used recursively to build a ML-hGRN in layer-by-layer fashion until the defined number of layers was obtained or terminated automatically.

**Conclusions:**

We validated the algorithm and demonstrated its high efficiency in constructing ML-hGRNs governing biological pathways. The algorithm is instrumental for biologists to learn the hierarchical regulators associated with a given biological pathway from even small-sized microarray or RNA-seq data sets.

**Electronic supplementary material:**

The online version of this article (doi:10.1186/s12859-016-0981-1) contains supplementary material, which is available to authorized users.

## Background

Present knowledge indicates that genes in genomes operate in multilayered hierarchical gene regulatory networks (ML-hGRNs) to control biological processes and pathways [1–6]. A typical hGRN contains a few high hierarchical regulators, some middle-level regulators and many terminal/structural genes at bottom layer. Studies have shown high hierarchical regulators seem to be global modulators that respond to various cellular signals [7, 8] and environmental cues [3, 9]. The middle-level genes play the manager-like roles, through which the commands from high hierarchical regulators at upper layers are synthesized and then passed down to terminal genes at bottom layer for execution [3, 10]. In general, the high hierarchical regulators at the top levels have more pleiotropic effect while terminal or structural genes have more specific functions. Genes involved in various metabolic or canonical pathways are regulated by ML-hGRNs [11, 12]. To understand how pathways are regulated, we should develop methods for constructing ML-hGRNs that operate above biological pathways and processes. This kind of ML-hGRNs can provide a hierarchy in addition to connectivity of regulators, which are essential in understanding wired complex regulation on metabolic or canonical pathways through multiple chains-of-command [13].

Despite the critical importance of ML-hGRNs, there is a lack of computational algorithms for directly building ML-hGRNs from gene expression data. Reverse engineering of ML-hGRN governing a biological pathway or process remains challenging, and the algorithms that are well suited for building ML-hGRNs have not yet been established. Although several algorithms have been developed for reverse-engineering GRNs, they are not specifically tailored for constructing the ML-hGRNs that mimic the hierarchical regulation [10]. Currently, the majority of gene expression data in public repositories are static data, namely, non-time-course data or time course data with large time intervals that vary from a few hours to even several days [14]. These types of data often miss some regulatory events and interactions between two adjacent time intervals, nullifying dynamic methods that include differential equation [15], finite state [16], dynamic bayesian network [17], control logic [18], boolean network [19], and stochastic networks [20]. In general, the methods that are of more useful to static data include various static methods that comprise GGM [21], mutual information based-RN [22], Algorithm for the Reconstruction of Accurate Cellular Networks (ARACNE) [23], Context Likelihood of Relatedness (CLR) [24], C3NET [25], MI3 [26], and probabilistic based--Bayesian network [27]. Bayesian network, a probabilistic graphic model that represents a set of variables and their conditional dependencies via a directed acyclic graph, has been used to infer the “optimal” structure of a set of genes. However, due to astronomical number of possible structures and the computational complexity, approximate inference methods, such as Gibbs sampling, Metropolis–Hastings algorithms and other variant methods, instead of the original probability method of Bayesian network, were used to approximate the inference of the possible structures of GRNs [27], and these methods are capable of producing a local interacting dependence variables containing causality relationships. ARACNE is the other widely used algorithm for building graphic dependency network using mutual information-based method. It can identify both linear and non-linear dependence relationships between genes while eliminating potential indirect gene–gene interactions through implementing data processing inequality (DPI). Another information-theory-based algorithm is the Context Likelihood of Relatedness (CLR), which scores all possible pairwise interactions based on mutual information and compared that to an interaction specific background distribution. Although computationally less complicated, most of the methods that evaluate pairwise relationships among genes can easily lead to spurious relationships when the number of RNA-seq or microarray data sets is small. Finally, GGM uses the partial correlation as a measure of dependency and interaction between any pair of genes by removing effect of third one [28], allowing to distinguish direct from indirect interactions [4]. Recently, GGM with a limited-order partial correlation function, which estimates correlations conditional on one or two, but not all other genes, and has been used to infer gene networks from *Arabidopsis* [29] and yeast [30] transcript profiles. However, as aforementioned, these methods are not specifically designed and tuned for inferring ML-hGRNs.

Although mathematical models are critically important in capturing the causality relationships for reconstructing gene networks, what is also very important is the biological regulatory models, which, when integrated into mathematical models, can empower efficiency of the network construction algorithms substantially. Biological regulatory model, in this study, refers to the defined regulatory structure to which the input genes can be functionally fitted in and then evaluated as a building block during network construction process. The integration of a biologically valid regulatory model into an algorithm demands seamless design that can enhance the recognition of the causal regulatory relationships of input genes. In this study, we developed a novel algorithm, named as bottom-up GGM algorithm, specifically for reverse engineering of ML-hGRNs that govern a biological pathway or process through integration of GGM algorithm with an authentic biological regulatory model. The input files for bottom-up GGM algorithm include: (1) the transcriptomic profiles of differentially expressed genes (DEGs) involved in a known metabolic pathway, or a canonical pathway defined by a gene ontology (2) the transcriptomic profiles of differentially expressed transcription factors (TFs) or all TFs under experimental condition. We evaluated bottom-up GGM algorithm for several pathways or biological processes and found it in general performs well for constructing ML-hGRNs. We believe the algorithm can meet the great needs for constructing ML-hGRNs using small- to medium-sized gene expression data sets, and the ML-hGRNs built will be instrumental for us to understand the hierarchical regulation of many biological processes and pathways.

## Results

### Selection of genes for bottom-layer and top regulatory layers

We applied our bottom-up GGM algorithm to multiple pathways for reverse-engineering ML-hGRNs, each is considered to govern a given pathway or biological process. For each pathway, there were two files: one contained the gene expression profiles of pathway genes of interest, and the other file contains the expression profiles of all TF genes. The genes involved in a pathway are generally non-regulatory genes,, which can be obtained from existing annotation of metabolic pathways. Certainly, genes involved in a biological process, for example, as defined by a gene ontology that is enriched in differentially expressed genes, can be treated as a canonical pathway, and used to replace the pathway genes.

### Reverse-engineering of ML-hGRN governing lignocellulosic pathway

The plant lignocellulosic pathway controls the biosynthesis of wide variety of secondary metabolic compounds including cellulose and lignin [31]. In addition to their roles in the structure and protection of the plants, cellulose and lignin have important roles in the structural integrity of plant cell walls, and the stiffness and strength of stems [32, 33].

**Fig. 1:**
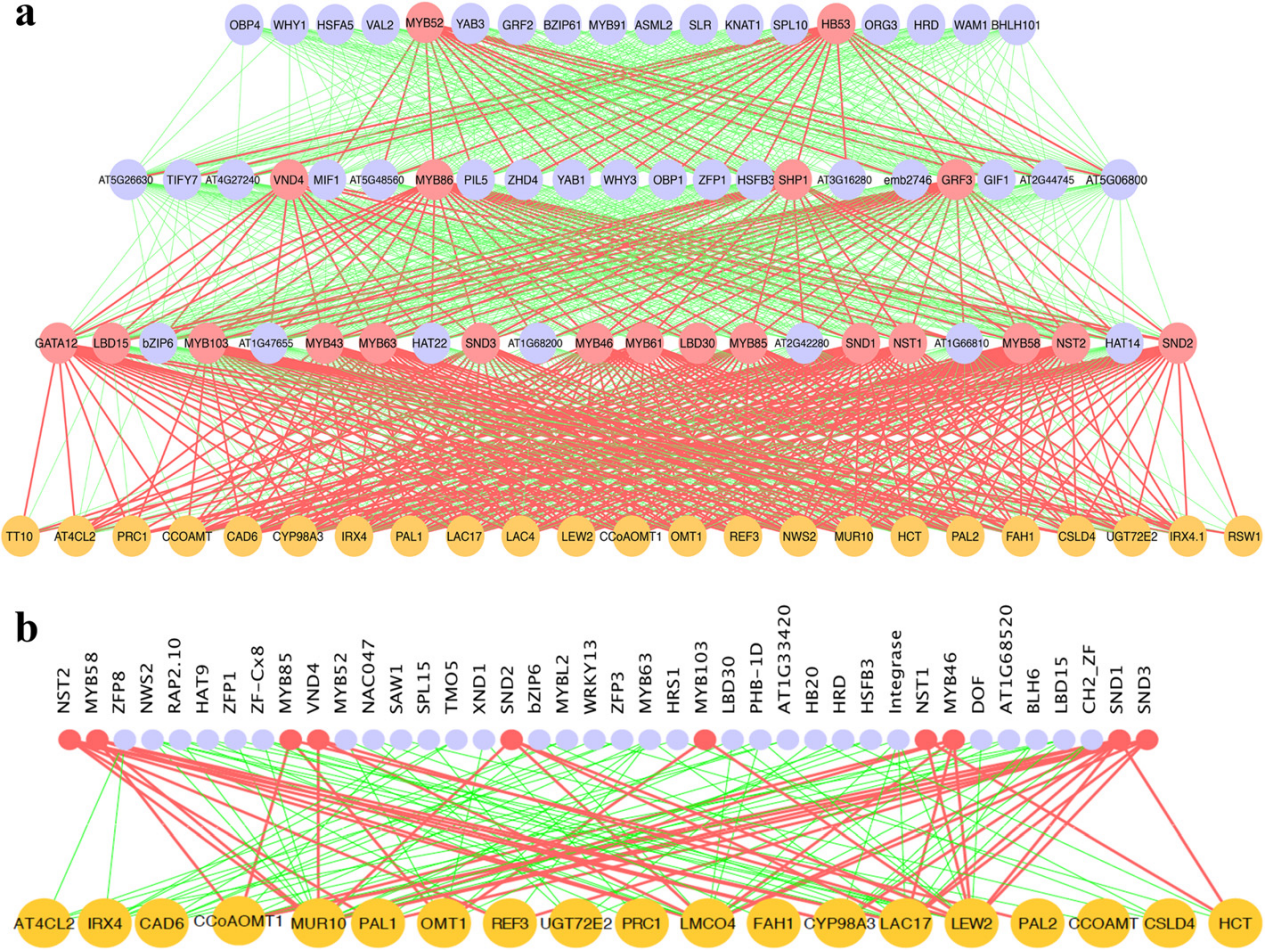
Construction of ML-hGRN for lignocellulosic pathway. **a**. Four-layered hGRN built with GGM bottom-up algorithm. The gene IDs represented by each symbol can be found in Additional file 1: Table S1. **b**. The gene association network of lignocellulosic pathway built with ARACNE algorithm that identifies expression profile-dependent genes. The input files for both bottom-up GGM algorithm and ARANCE include the profiles of 1622 transcription factors and 25 lignocellulosic pathway genes. The nodes with coral (red) color highlight in both networks are known regulatory TFs for lignocellulosic pathway in existing knowledgebase. The gene IDs represented by each symbol can be found in Additional file 1: Table S2

The inputs for our bottom-up GGM algorithm include the profiles of 25 pathway genes, and 1622 TFs extracted from the 128 pooled *Arabidopsis* microarray data sets under short-day condition that is known to induce secondary wall biosynthesis. We constructed a ML-hGRN using these 25 pathway genes used as bottom layer and these TFs as candidates for top layers. The construction of ML-hGRN was a dynamic process in our pipeline software with the parameters predetermined by users. These parameters include the number of layers, significant levels of p values for correlation and partial correlation and their differences, and a percentage of genes to be kept for each layer above the bottom one. The pipeline first built the second layer immediately above the bottom (or first) layer, and then used the second layer as the bottom layer, and repeated the above procedure to obtain the third layer and so on. For this ML-hGRN (Fig. 1a), we obtained 14, 16, and 18 TFs for second, third and fourth layer respectively above the bottom layer. The detailed results that include correlations and p-values for all the interfering TFs are given in (Additional file 1: Table S1). There are 14 TFs in the second layer (Fig. 1), out of which 10 TFs (*GATA12*, *SND1*, 2, and 3, *MYB85*, *NST1 and 2*, *MYB103*, *MYB46* and *MYB58*) were positive TFs known to regulate lignocellulosic biosynthesis [34]. NAC domain proteins: NST1 [35], NST2 [36], and SND1 (also called NST3) [35, 37] are key regulators of secondary wall biosynthesis. NST1, NST2 and NST3 are key regulators involved in wall thickenings in various tissues when expressed ectopically [36, 38]. The expressions of *SND2, SND3, MYB103*, and *MYB46* are regulated by SND1 and all are developmentally associated with cells undergoing secondary wall thickening [39–41]. The SND2 regulates genes involved in secondary cell wall development in *Arabidopsis* fibres, and increases fibre cell area in *Eucalyptus* [42]. MYB46 regulates the biosynthesis pathways of cellulose, xylan, and lignin [40] and *GATA12* [43] controls xylem vessel differentiation. In the third layer, there are 6 positive TFs (MYB43, *MYB92, MYB61, MYB63, MYB86, GRF3*). The MYB63 is known to be involved in the activation of lignin biosynthetic pathway during secondary wall formation in *Arabidopsis*. The TF MYB61 controls stomatal aperture in *Arabidopsis* [44] and is required for mucilage deposition and extrusion in the seed coat [45]. MYB84 regulates the accumulation of the UV-protectant compound sinapoylmalate by repressing the transcription of the gene encoding the lignocellulosic enzyme cinnamate 4-hydroxylase [46], and the *MYB43* regulates the thickening of secondary wall of cells [47]. In fourth layer, there are 18 TFs. Most appear to be the high hierarchical regulators. Some of them are known to be responsive to various environmental cues and intercellular cues, for example, SLR [48] and HB53 [49] are auxin-inducible whereas ERF38 [50] and OBP3 [51] are responsive to ethylene and salicylic acid, respectively. In this layer, the SHP1, also referred as to be SHATTERPROOF1, is known to control the differentiation of the dehiscence zone where it promotes the lignification of adjacent cells [52] while HB53 boosts vascular development in meristem [53].

Although we have indicated there are currently no computational algorithms for directly building ML-hGRNs from gene expression data, ARANCE can take the same inputs as our bottom-up GGM algorithm and obtain TFs that have dependency on pathway genes. These TFs can serve as rough controls and allow us to obtain some idea of the performance of our bottom-up GGM algorithm. We input these expression profiles of 25 pathway genes and 1622 TFs to ARANCE, and obtained the TFs that have dependency with at least two pathway genes with mutual information > 0.25 (Additional file 1: Table S2). Of the 40 TFs obtained, there are 13 positive TFs (Fig. 1B). Ten of them are common with the ML-hGRN built with bottom-up GGM algorithm. Although bottom-up GGM failed to capture three TFs, *VND4, MYB52* and *XND1*, which were recognized by ARANCE, it identified eight more positive TFs that included *GATA12, MYB86, MYB43, MYB61, GRF3, MYB92, SHP1*, and *HB53*.

### Construction of ML-hGRN that controls human embryonic pluripotency renewal

**Fig. 2:**
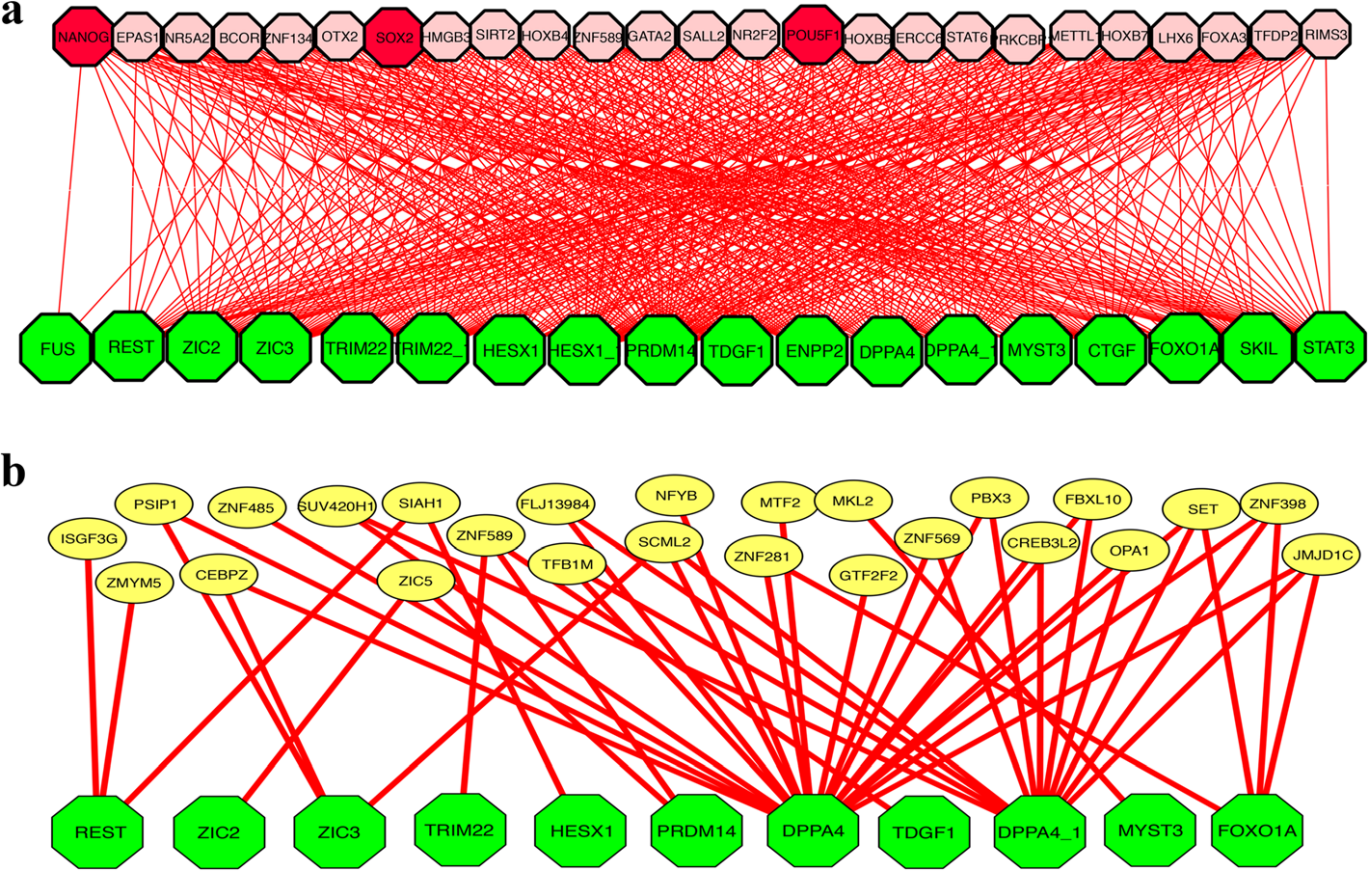
Construction of ML-hGRN for putative pluripotency renewal pathway in human embryonic stem cells. **a**. Two-layered hGRN built with bottom-up GGM algorithm. The gene IDs represented by each symbol can be found in Additional file 1: Table S3. **b**. The network built with ARACNE algorithm. The input files for both bottom-up GGM algorithm and ARANCE include the expression profiles (microarrays) of 2189 transcription factors and 19 putative pathway genes identified from literature [54]. Only one layer was built and red nodes in the second layer represent the three master transcription factors known to control the pluripotency renewal. The gene IDs represented by each symbol can be found in Additional file 1: Table S4

There are three master transcription factors, NANOG, POU5F1, and SOX2, which are known to govern the pluripotentcy renewal in human embryonic stem cells [54]. Early studies have identified some target genes that can be bound by the above three transcription factors using ChIP-seq experiments [54, 55]. We assume that these target genes belong to a canonical pathway that plays key roles in pluripotency renewal. We would like to test if we could infer these three master transcription factors by building a one-layered hGRN using our bottom-up GGM algorithm. The 189 microarray data sets [56, 57] for human stem cells was collected from 17 experiments in which hES cells were treated with various differentiation reagents. Therefore, these datasets include states involved in many regulatory events underpinning pluripotency, such as ES maintenance, exiting the pluripotent state, and differentiation. We used 19 target genes as bottom-layer, all TFs in human as inputs, and then used our bottom-up GGM algorithm to build one regulatory layer above these 19 pathway genes. The network we obtained is shown in Fig. 2a, with 25 top genes shown in second layer. All above three transcription factors were shown up in top 25 TFs captured (2*λ* was used). When the same inputs were used for ARANCE, and the network obtained is shown in Fig. 2b. We also kept top 25 TFs based on the mutual information (MI) on the second layer but none of above three TFs was present in these 25 genes. We searched the rankings of above three TFs in the ARANCE output sorted by MI, and found SOX2 and NANOG ranked at 68 and 154, respectively.

### Implementation of the bottom-up GGM to synthetic yeast data

We generated a set of synthetic compendium gene expression data sets using the SynTReN software [58] and the regulatory network models based upon yeast experimental data as original seeds. There are 200 genes each with 100 expression values. From these 200 genes, we randomly selected 48 non-regulatory genes, which are presumably the pathway genes, and were used as bottom layer. The remaining genes are regulatory genes that contained 26 positive TFs. The results are shown in Additional file 1: Table S5. We constructed only one layer in order to make a better comparison with the result generated with ARCNE method. The list of top 50 TFs yielded from bottom-up GGM algorithm contains 25 the positive TFs, whereas, the top 50 TFs yielded from ARANCE contain 15 positive TFs, indicating the evaluation of triplet genes for causal relationships may have some advantages over pairwise evaluation used by ARANCE.

### Performance comparison between the networks constructed by bottom-up GGM and ARACNE

**Fig. 3:**
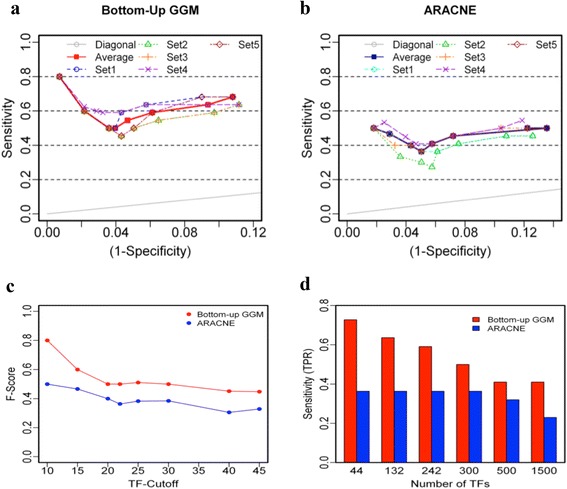
The efficiency of bottom-up GGM algorithm. **a**. ROC curves of bottom-up GGM algorithm resulted from five testing data sets, each contains 300 TFs, and 25 pathway genes. **b**. ROC curves of ARACNE resulted from five testing data sets, each contains 300 TFs, and 25 pathway genes. **c**. F scores of bottom-up GGM and ARACNE in terms of different TF-cutoffs. **d**. The relationship between true positive rates (TPR) and different numbers of TFs as inputs. The TPR of ARACNE is uniform because it captured just one positive for various numbers of TF inputs varying from 44 to 1500

We ran bottom-up GGM and ARANCE algorithm with the 25 wood formation genes as bottom layer, and five groups of 322 TFs as candidates for regulatory layer. Based on the number of positive TFs that were built-in regulatory layer, we calculated the sensitivities and specificities for eight different numbers of TF cutoffs, namely, the number of TFs retained in the second layer. For ARCNE, we just kept those TFs that were directly dependent on wood formation genes and counted their frequencies. After the same TF cut-offs as bottom-up GGM algorithm were applied, we calculated the sensitivities and specificities. For both bottom-up GGM and ARACNE, we plotted ROC curves (sensitivity vs (1-specificity)) (Fig. 3a and b). The dashed curves shown in Fig. 3 (a, and b) correspond to the five groups of TF inputs and the solid curve is the average of the five curves. The cohesion of dashed curves suggests that the performance of our algorithms was persistent and did not change much with different groups of TFs. We also calculated the F-scores from the averaged curves and plotted against the different TF cutoffs for both bottom-up GGM algorithm and ARANCE (Fig. 3c). Higher F-score represents better performance. To see how the performance of the bottom-up GGM algorithm changes with the size of the data set (number of genes in the input), we calculated and plotted the True Positive Rates (TPR) against different number of TFs in the input for same TF cutoffs (Fig. 3d). We used 22 TF cutoff to find the TPR in Fig. 3d as there are 22 positive TFs in the input file. It is obvious that the bottom-up GGM has significantly higher TPR than ARACNE.

## Discussion

Like GGM, we have used the first order partial correlation but adopted a biological and mathematical model integrated approach to infer ML-hGRNs. Our approach is based on a biological theory that when a TF exerted its control over a pair of genes, the correlation between these two genes will be either enhanced or impaired. The change in correlation is represented by a significant difference between the correlation of the pair of genes (in the presence of a regulatory TF, z), namely, *r*_*xy*_, and the correlation of the same pair of genes at the absence of this TF, namely *r*_*xy*| *z*_. We implemented a multivariate delta method [59] to test the significance of difference *d* = *r*_*xy*_ − *r*_*xy*| *z*_ in four different circumstances where the null hypothesis of zero difference. The higher accuracy of method can be at least partially ascribed to the integration of biological triplet gene regulatory model, which played significantly roles in the following aspects: (1) gene (variable) reduction. By fitting differentially expressed regulatory genes and a group of pathway genes into the model based on their annotation, the dimensionality of gene space can be significantly reduced. (2) noise reduction. By filtering out the irrelevant genes that do not fit the biological model, we significantly reduced the noise, enabling the true regulatory relationships to be easily emerged. (3) reduction in gene dimensionality and explicitly defined roles of input genes in turn empowered mathematical modeling for capturing true regulatory relationships. In addition to the integration of biological regulatory model that enhanced the efficiency of the approach, the association of two paired genes under same regulatory mechanism was achieved by Spearman method, a non-parametric method that measures the strength of association between two ranked variables, which was demonstrated to be extremely well-suited for associating functionally relevant genes as compared to seven other gene association methods [57]. Spearman correlation method makes no assumption about the distribution of the data, and Spearman rho measures the strength of linear/non-linear monotonic relationship between two variables [57]. In addition, the bottom-up GGM algorithm performs exhaustive combinations of genes, providing numerous evaluation opportunities for positive genes to be eventually emerged from large number of candidates. Finally, we integrated weighted sparse canonical correlation analysis method (WSCCA) to determine the number of genes being kept for each layer, which in general could produce ideal number of regulators for each layer. To identify the TFs with higher interference frequency to pathway genes, one can double the *λ* value.

The efficiency of triplet gene model utilized in this study was experimentally validated in our earlier study [4]. In that study, we used a probability model and triple gene model combined approach to build a SND1-mediated two-layered hGRN in a top-down fashion with 12 RNA-seq libraries as input. Up to 97 % regulatory relationships in the built network were successfully proven by ChIP-PCR using SND1 antibodies and again verified by RT-PCR in stable transgenic lines where these targets were activated by overexpressed SND1 [4], indicating the efficiency of triplet gene regulatory model. In addition, the early version of this bottom-up GGM algorithm was implemented to nine RNA-seq libraries generated from nine independent poplar overexpression transgenic lines of microRNA397a [5]. microRNA397a targets mRNAs species of different laccase genes whose proteins catalyze polymerization reactions of S, G, and H monomers during lignin biosynthesis [5]. We constructed a three-layered hGRNs with 14 differentially expressed laccase genes as bottom-layer. The algorithm successfully identified microRNA397a from 1208 regulatory genes and constructed it into the secondary layer, where it directly regulated 12 laccase genes at the bottom layer. We chose seven laccase genes to validate using 5’RACE, and five of them were proven to be down-regulated in the microRNA397a transgenic lines. In the network built, several transcription factors that were known to govern lignin biosynthesis were recognized by our triple gene model and built into the regulatory layers. These experimental results implicate that the networks built from our bottom-up GGM algorithm are highly accurate and trustworthy.

Finally, the bottom-up GGM algorithm we developed can be used to obtain a hierarchy of TFs that are coordinated to pathway genes in expression profiles at the bottom layer. We believe these hierarchical TFs contain significantly enriched positive genes that govern the underlying pathway either directly or indirectly, allowing biologists to initiate the experimental validation. Our method is compliant with the present knowledge that when the gene expression profile of a transcription factor and the profile of target gene are correlated, it is more likely that the target genes are authentic targets [60, 61]. We employed robust Spearman-rank correlation that has been proven to be efficiently in associating loosely and functionally coordinated genes as shown earlier [57] to augment the recognition of real hierarchical TFs that collectively control underlying pathway genes. The computational validation with test data sets has shown the positive regulatory genes can be significantly enriched in the built ML-hGRNs. We believe the algorithm is instrumental for constructing the hGRNs that govern pathways or biological processes.

## Conclusions

A bottom-up graphic Gaussian model (GGM) algorithm was developed for constructing a multilayered hierarchical gene regulatory network that operates above a given metabolic or canonical pathway using small- to medium-sized microarray or RNA-seq data sets. The algorithm was validated with both synthetic and real gene expression data sets, leading to the networks that were dominated with significantly enriched known positive regulatory genes in most of the cases. We believe the algorithm is in particular instrumental for biologists to identify the hierarchical regulators associated with a given biological pathway of interest for experimental validation.

## Methods

### Arabidopsis and human microarray data sets

The *Arabidopsis* gene expression data used in this study were downloaded from public repository. The wood formation compendium data set contains the 128 microarrays pooled from six experiments, which have the accession identifiers of GSE607, GSE6153, GSE18985, GSE2000, GSE24781, and GSE5633, in NCBI Gene Expression Omnibus(GEO) (http://www.ncbi.nlm.nih.gov/geo/). These data sets were obtained from hypocotyledonous stems under short-day that can induce secondary wood formation [62]. The salt stress compendium microarray data set contains108 microarrays from 6 experiments with the accession identifiers of GSE5620, GSE6153, GSE24781, GSE5633, GSE6151, GSE18985) in the NCBI GEO database. This salt compendium data were used in our earlier research [56, 63]. All data sets mentioned above were derived from hybridization of Affymetrix 25 k ATH1 microarrays. The original CEL files were downloaded and processed by the robust multiarray analysis (RMA) algorithm using the Bioconductor package. For quality control we used the methods that were previously described [64]. The 189 human microarray data sets were introduced in detail in our previous publication [56].

### Biological model for reverse-engingeering ML-hGRNs

Based on the fact that genes with similar patterns of transcriptomic expression are likely to be regulated via same mechanism [65–67], we proposed that when a regulatory gene in layer *i +1* of a hGRN controls a pair of genes in layer *i, where i* ≥ 0, the presence of this regulatory gene either significantly enhances or impairs the correlation of the paired genes at one level below, we consider this regulatory gene interfere with the paired genes. This model was integrated into the bottom-up GGM algorithm to evaluate the building blocks of combined triple genes, namely, a regulatory gene in layer *i +1 and* a pair of genes in layer *i*, during the construction of ML-hGRN using GGM.

### Biological model based graphic Gaussian model (GGM) for construction of ML-hGRNs

**Fig. 4:**
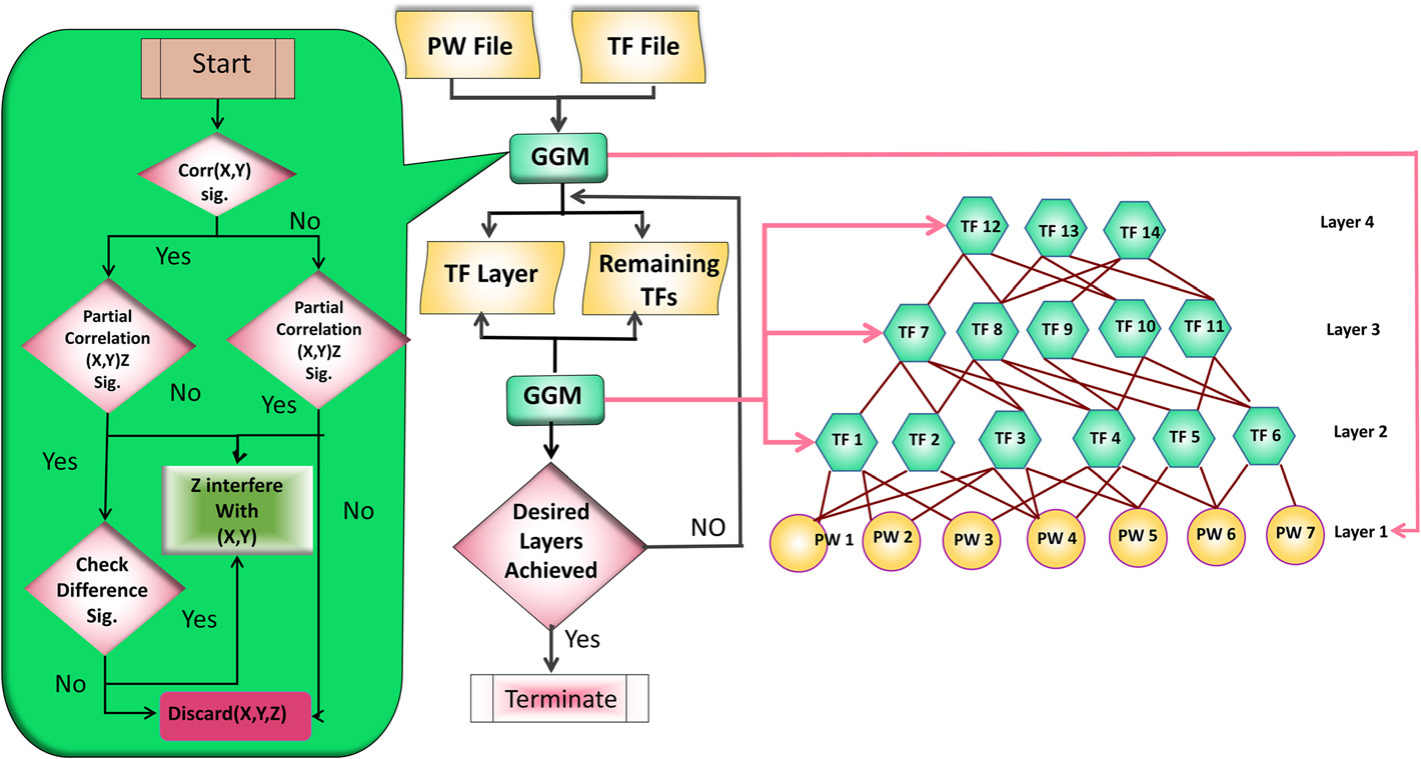
The flowchart illustrating the procedure of bottom-up GGM algorithm using a group of pathway genes and all regulatory genes or a subset of regulatory genes that are significantly altered under experimental condition

We developed a bottom-up GGM algorithm that contains multi-step mathematical procedure to construct ML-hGRN operating above a biological process or pathway. The algorithm integrated the GGM with the biological model as described earlier. Initially, the pathway genes were placed at bottom layer, namely layer *i =1*, while regulatory genes like TFs were used as candidate genes for layer *i* +1. The algorithm evaluated each combination of triplet genes, namely one regulatory gene, and two pathway genes, to determine if the regulatory gene significantly interfered with the two pathway genes. The interference of regulatory gene on two pathway genes could enhance or impair the coordination of two pathway genes. The significance of interference was tested by examining if the significant difference existed between the correlation of two pathway genes in the presence of the regulatory gene, namely, *r*_*xy*_, and the correlation of the same pair of genes at the absence of this TF, namely *r*_*xy*| *z*_. We implemented a multivariate delta method [59] to test the significance of difference *d* = *r*_*xy*_ − *r*_*xy*| *z*_ in four different circumstances where the null hypothesis of zero difference. The four circumstances shown in Fig. 4 are: (1) The correlation is significant but the partial correlation is not significant, indicating that the presence of the TF make the paired pathway genes more coordinated. Therefore, the TF interfered with the pathway genes pair. (2) Both the correlations are significant. The pathway genes in the pair are correlated before and after removing the effect of the transcription factor. To find if TF has significant effect on the relation of the two pathway genes, we need to determine if the difference between the correlations and partial correlation are significant. (3) The correlation is not significant but partial correlation is significant, which implies the TF is interfering. (4) Both correlation and partial correlation are not significant. In this case, we discarded the triplet and moved to the next triplet genes. Both correlation and partial correlation were calculated using Spearman Rank Correlation method. Spearman method is one of the most effective method to identify functionally associated genes using microarray data [57]. The pseudo code for the bottom-up GGM-algorithm is shown below.

**Pseudo code** for regulatory model based statistical model

1. Input: (*G*, *R*) [*G* → BPP genes, *R* → TFs], where BPP represents biological pathways or processes
2. For each pair (*x*, *y*), [(*x*, *y*) ∈ *G*]
  Calculate pairwise correlation of (*x*, *y*), *r*_*xy*_ = *cor*(*x*, *y*), and partial correlation *r*_*xy*|*z*_ = *pcor*(*x*, *y*|*z*) (partial correlation of *x*, *y* given z∈ *Z*).
3. Perform the significance test for the correlation and the partial correlation and find the corresponding p-values.
4. Find the difference of the correlations and perform the significance test:
  Difference of correlations: $D={r}_{xy}-\frac{r_{xy}-{r}_{zx}{r}_{zy}}{\sqrt{\left(1-{r}_{zx}^2\right)\left(1-{r}_{zy}^2\right)}}$
  Standard error of *D* ≜ *D*_*se*_
  Find the test statistics, ${z}_1=\frac{D}{D_{se}}$, which follows a standard normal distribution approximately.

The difference of correlation, *D*, is a function of three correlations, *r*_*xy*_, *r*_*yz*_ and *r*_*xz*_, and denote *D* = *f*(*r*_*xy*_, *r*_*yz*_, *r*_*xz*_). Let

$$ {\displaystyle {\sum}_r = \left[\begin{array}{ccc}\hfill {\sigma}_{11}\hfill & \hfill {\sigma}_{12}\hfill & \hfill {\sigma}_{13}\hfill \\ {}\hfill {\sigma}_{21}\hfill & \hfill {\sigma}_{22}\hfill & \hfill {\sigma}_{23}\hfill \\ {}\hfill {\sigma}_{31}\hfill & \hfill {\sigma}_{32}\hfill & \hfill {\sigma}_{33}\hfill \end{array}\right]} $$

be the covariance matrix of (*r*_*xy*_, *r*_*yz*_, *r*_*xz*_).

Let (*pd*_1_, *pd*_2_, *pd*_3_) denote the partial derivatives $\left(\frac{\partial D}{\partial {r}_{xy}},\ \frac{\partial D}{\partial {r}_{zy}},\frac{\partial D}{\partial {r}_{xz}}\right)$ of *D* with respect to (*r*_*xy*_, *r*_*yz*_, *r*_*xz*_), then according the multivariate Delta method [59], the variance of *D*, is approximated by

$$ var(D)\approx {\displaystyle \sum_{i=1}^3}{\displaystyle \sum_{j=1}^3}p{d}_i{\sigma}_{ij}p{d}_j. $$

The standard error is taken as the square root of *var*(*D*). That is, ${D}_{se}=\sqrt{var(D)}$.

The formulae to calculate the variance and covariance among the correlations were based on asymptotic theory [68].

$$ \begin{array}{l}{\sigma}_{11}=var\left({r}_{xy}\right)=\frac{{\left(1-{r}_{xy}^2\right)}^2}{n}\\ {}{\sigma}_{22}=var\left({r}_{yz}\right)=\frac{{\left(1-{r}_{yz}^2\right)}^2}{n}\\ {}{\sigma}_{33}=var\left({r}_{xz}\right)=\frac{{\left(1-{r}_{xz}^2\right)}^2}{n}\\ {}{\sigma}_{12}=cov\left({r}_{xz},{r}_{xy}\right)=\frac{\left(2{r}_{zy}-{r}_{xz}{r}_{xy}\right)\left(1-{r}_{zy}^2-{r}_{xz}^2-{r}_{xy}^2\right)+{r}_{zy}^3}{2n}\\ {}{\sigma}_{13}=cov\left({r}_{xz},{r}_{zy}\right)=\frac{\left(2{r}_{xy}-{r}_{xz}{r}_{zy}\right)\left(1-{r}_{zy}^2-{r}_{xz}^2-{r}_{xy}^2\right)+{r}_{xy}^3}{2n}\\ {}{\sigma}_{23}=cov\left({r}_{zy},{r}_{xy}\right)=\frac{\left(2{r}_{zx}-{r}_{zy}{r}_{xy}\right)\left(1-{r}_{zy}^2-{r}_{xz}^2-{r}_{xy}^2\right)+{r}_{zx}^3}{2n}\end{array} $$

The partial derivatives of the difference are

$$ \begin{array}{l}p{d}_1=1-\frac{1}{\sqrt{1-{r}_{zy}^2}*\sqrt{1-{r}_{xz}^2}}\\ {}p{d}_2=\frac{\left({r}_{xz}-{r}_{xy}*{r}_{zy}\right)}{\Big(\left(\sqrt{1-{r}_{xz}^2\Big)}*{\left(1-{r}_{zy}^2\right)}^{1.5}\right)}\ \\ {}p{d}_3=\frac{\left({r}_{zy}-{r}_{xy}*{r}_{zx}\right)}{\Big(\left(\sqrt{1-{r}_{zy}^2\Big)}*{\left(1-{r}_{zx}^2\right)}^{1.5}\right)}\ \end{array} $$

### Bottom-up GGM algorithm for reconstruction of ML-hGRNs controlling a biological pathway or biological process

Based on the regulatory model and mathematical model as described, we developed the following integrated procedure to reconstruct ML-hGRNs using two inputs: the profiles of pathway genes, and the profiles of TFs. These pathway genes and TFs are in general from differentially expressed gene sets under the experimental condition in which profiles were obtained.

1. Initially, the algorithm set a group of pathway genes to be the bottom layer, and built regulatory layer immediately above the bottom layer.
2. Using the aforementioned GGM method as shown in **Pseudo code** for regulatory model based statistical model, we evaluated the relationships between each paired pathway gene pair from the bottom layer with a TF from TF pool. When a TF interfered (either enhanced or impaired) the coordination of two paired genes with significant p-values, keep them. Discard this TF otherwise. Continue until all TFs are evaluated.
3. When a given pathway gene pair against all TF are completed, pick up a new pair of genes from bottom layer. Repeat Step 2 and 3 until all pair of bottom-layer genes against all TFs are completed.
4. Correct the interfering p-values for multiple testing and keep the triple genes in which the TFs and two paired bottom-layer genes have corrected p-values less than the given significance level.
5. Count the total number of paired genes each TF interferes with.
6. Sort the TFs by the number of significantly interfered genes from the largest to smallest and determine the number of TFs of interest to be retained in current layer using the method given below.
7. Remove the TFs kept in current layer from the TF input file, and then set the current layer as bottom layer, and then repeat step 1-6 with the remaining TFs to obtain a new layer.
8. We repeat the above steps from 2-7 until the desired number of layers is obtained.

This framework enhances the discovery of regulatory layers operating over a pathway or a biological process by recursively evaluating and identifying candidate regulatory genes with strongest interference on layer in a layer-by-layer fashion.

### Determination of TF interference on paired target genes

In the step 3 of above-mentioned procedure, we need to determine how TF interferes two paired genes. Let *p*_1_ and *p*_2_ be the p-values of the significance tests of correlation and partial correlation respectively. If *r*_*xy*_ and *r*_*xy*|*z*_, are not significant i.e., the p-values *p*_1_ and *p*_2_ are greater than the significance level, discard (*x*, *y*, *z*). If *r*_*xy*_ is significant and *r*_*xy*|*z*_ is not significant i.e., *p*_1_ is less and *p*_2_ is greater than the given significance level then *z* interferes with *x* and *y*. If both, *r*_*xy*_ and *r*_*xy*|*z*_ are significant then test the significance of the difference between *r*_*xy*_ and *r*_*xy*|*z*_. Let *p*_3_ to be the p-value of the test. If there is significance difference between the correlations i.e., if *p*_3_ is less than the pre-specified significance level, then *z* interferes with *x* and *y*. There is no interference of *z* otherwise. If *p*_1_ is greater but *p*_2_ is less than the given significance level, then, *z* interferes with *x* and *y*.

### Determination of number of TFs to be kept in each layer

In order to determine how many TFs should be included in each layer, we designed a weighted sparse canonical correlation analysis method (WSCCA), which is similar to Witten’s sparse canonical correlation analysis method (SCCA) [69]. For the construction of first layer network, let *X*_*n* × *p*_ be the pathway gene expression matrix, where *n* denotes the number of samples and *p* denotes the number of pathway genes. Let *Y*_*n* × *q*_ be the expression matrix of TFs we get in step 6 of above given algorithm, where *q* denotes the number of TFs. The matrices, *X* and *Y* are centered and scaled. Canonical correlation analysis (CCA), developed by Hetelling [70], involves finding vectors *u* and *v* that maximize *cor*(*Xu*, *Yv*), that is

$$ maximiz{e}_{u,v}{u}^T{X}^TYv\kern0.5em  subject\ to\ {u}^T{X}^TXu=1,\ {v}^T{Y}^TYv=1 $$

There is a closed-form solution for canonical vectors *u* and *v*. However, *u* and *v* are not sparse, and these vectors are not unique if *p* or *q* exceeds *n*. SCCA use *l*_1_ penalization for high-dimensional problems to get sparse *u* and *v*. So the optimization problem now is

$$ maximiz{e}_{u,v}{u}^T{X}^TYv+{\lambda}_1\left|\right|\kern0.1em u\kern0.1em \left|\right|{}_1+\kern0.5em {\lambda}_2\;\left|\right|\kern0.1em v\kern0.1em \left|\right|{}_1\kern0.5em  subject\ to\kern0.75em \left|\right|\kern0.1em u\kern0.1em \left|\right|{}_2^2=1,\ \left|\right|\kern0.1em v\kern0.1em \left|\right|{}_2^2=1 $$

In our setting, as the number of pathway genes is not big, there is no need to impose *l*_1_ penalization on vector *u*. We used the number of interference of each TF as weight to penalize vector *v*, our optimization model is

$$ maximiz{e}_{u,v}{u}^T{X}^TYv+\lambda \kern0.1em \left|\right|\kern0.1em {W}^Tv\kern0.1em \left|\right|{}_1\kern0.5em  subject\ to\ \left|\right|\kern0.1em u\kern0.1em \left|\right|{}_2^2=1,\ \left|\right|\kern0.1em v\kern0.1em \left|\right|{}_2^2=1 $$

Where the *i*^*th*^ element of weight vector *W* is maximal number of interference minus the number of interference of *TF*_*i*_. Then the above model becomes a biconvex problem. We designed the following algorithm to solve it.

1. Initialize *v* to have *l*_2_ norm 1.
2. Iterate until convergence:
  - Fix *v*, solve *u* ← *ar gmx*_*u*_*u*^*T*^*X*^*T*^*Yv subject to* || *u* ||_2_^2^ = 1.
  - Fix *u*, solve *v* ← *ar gmx*_*v*_*u*^*T*^*X*^*T*^*Yv* + *λ* || *W*^*T*^*v* ||_1_ *subject to* || *v* ||_2_^2^ = 1.

The tuning parameter *λ* is determined by cross-validation. We keep the TFs selected by this algorithm in the current layer.

### Testing efficiency of the methods

We performed sensitivity, specificity and F-score analyses to assess the efficiency and quality of our algorithm. First, we built a test data set with the 129 microarray data sets, and 108 microarray data sets we used earlier [63]. The 25 pathway genes we used for initiating the ML-hGRN construction was the same as those used for building ML-hGRN governing lignocellulosic pathway and to construct the upper layers, 1500 TFs including 22 true positive TFs that are known to govern lignocellulosic pathway were used. We classified the 1500 TFs into five groups for testing the efficiency of the bottom-up GGM algorithm. Each group contained 278 randomly selected TFs from 1500 TFs. We then added 22 positive TFs to each group. These 300 TFs, together with the 25 wood formation genes, were used as inputs for building one layer of hGRN. We built one layer of hGRN and calculated the sensitivity specificity and F-score using the formulae as shown below. ARACNE was used for comparison.

1 $$ Sensitivity(TPR)=\frac{TP}{TP+FN} $$

2 $$ Specificity(SPC)=\frac{TN}{FP+TN} $$

3 $$ F- score=\frac{2\times Precision\times Recall}{\left( Precision+ Recall\right)} $$

Where,

4 $$ Recall= Sensitivity $$

5 $$ Precision(PPV)=\frac{TP}{TP+FP} $$

*TP*, *FP*, *TN*, and *FN* are true positive, false positive, true negative, and false negative respectively.

## Additional file

Additional file 1: Table S1. The output of ML-hGRN operating over lignocellulosic pathway built with the bottom-up GGM algorithm. **Table S2.** The output of regulatory layer over lignocellulosic pathway identified by ARACNE algorithm. **Table S3.** The ML-hGRN operating over human pluripotency renewal pathway built with the bottom-up GGM algorithm. **Table S4.** The output of regulatory layer over human stem cell renewal pathway identified by ARACNE algorithm. **Table S5.** Comparison of bottom-up GGM algorithm with ARANCE using yeast synthetic data. (XLSX 232 kb)
